# Directed evolution of peroxidase DNAzymes by a function-based approach

**DOI:** 10.1093/biomethods/bpae088

**Published:** 2024-12-13

**Authors:** Soubhagya K Bhuyan, Weisi He, Jingyu Cui, Julian A Tanner

**Affiliations:** School of Biomedical Sciences, LKS Faculty of Medicine, The University of Hong Kong, Hong Kong SAR, P.R. China; Advanced Biomedical Instrumentation Centre, Hong Kong Science Park, Shatin, Hong Kong SAR, P.R. China; School of Biomedical Sciences, LKS Faculty of Medicine, The University of Hong Kong, Hong Kong SAR, P.R. China; School of Biomedical Sciences, LKS Faculty of Medicine, The University of Hong Kong, Hong Kong SAR, P.R. China; School of Biomedical Sciences, LKS Faculty of Medicine, The University of Hong Kong, Hong Kong SAR, P.R. China; Advanced Biomedical Instrumentation Centre, Hong Kong Science Park, Shatin, Hong Kong SAR, P.R. China; Materials Innovation Institute for Life Sciences and Energy (MILES), HKU-SIRI, Shenzhen, P.R. China

**Keywords:** catalytic nucleic acids, DNAzymes, evolution, G-quadruplex, peroxidase DNAzymes, self-biotinylation reaction, self-biotinylated DNA

## Abstract

Peroxidase DNAzymes are single-stranded, stable G-quadruplexes structures that exhibit catalytic activity with cofactor hemin. This class of DNAzymes offers several advantages over traditional protein and RNA catalysts, including thermal stability, resistance to hydrolysis, and easy of synthesis in the laboratory. However, their use in medicine, biology, and chemistry is limited due to their low catalytic rates. Selecting and evolving for higher catalytic rates has been challenging due to limitations in selection methodology which generally use affinity as the selection pressure instead of kinetics. We previously evolved a new peroxidase DNAzyme (mSBDZ-X-3) through a directed evolution method, which was subsequently used for proximity labelling in a proteomic experiment in cell culture. Herein, we present a detailed protocol for this function-based laboratory evolution method to evolve peroxidase DNAzymes for future laboratory implementation. This approach is based on capturing self-biotinylated DNA, which is catalyzed by intrinsic peroxidase activity to select for DNAzyme molecules. The selection method uses fluorescence-based real-time monitoring of the DNA pools, allowing for the enrichment of catalytic activity and capture of catalytic DNA across evolutionary selection rounds. The evolved mSBDZ-X-3 DNAzyme attributes parallel G-quadruplex structure and demonstrates better catalytic properties than DNAzyme variants evolved previously. The influence of critical reaction parameters is outlined. This protocol enables discovery of improved peroxidase DNAzyme/RNAzyme variants from natural or chemical-modified nucleotide libraries. The approach could be applicable for the selection of catalytic activities in a variety of directed molecular evolution contexts.

## Introduction

G-quadruplex structures are highly polymorphic and stable under physiological conditions [1]. In nature, these structures do not exhibit peroxidase activity, likely due to the absence of the redox cofactor heme in their active sites. G-quadruplex DNAzymes were first discovered by serendipity when the PS2.M aptamer which binds to N-methyl mesoporphyrin IX (NMM) was found to have porphyrin metalation catalytic properties [2, 3]. Systematic evolution of ligands by exponential enrichment (SELEX) experiments led to the discovery of the PS2.M DNA aptamer based on its recognition and affinity for the NMM target. The selection process involved incubating a DNA library with NMM covalently attached to acrylic beads in a packed column. Unexpectedly, the PS2.M aptamer was observed to be capable to catalyze peroxidase reactions in complex with hemin in the presence of mild hydrogen peroxide [2, 3]. This discovery led to several further G-quadruplex DNAzymes reported for multiple biosensing applications [4–8].

Another alternative strategy for *in vitro* selection was performed to discover highly efficient G-quadruplex peroxidase DNAzymes [9]. This process utilized an agarose bead-based SELEX method to enrich hemin-binding aptamers from different percentages guanine-rich DNA libraries. Hemin was coupled to agarose beads through a reaction between the carboxyl and amine groups on the bead's surface. Subsequently, beads were used as a solid support for enriching positive binders from libraries based on binding affinity through multiple selection rounds. The final selection round of DNA sequences were amplified for cloning and characterization, ultimately leading to the discovery of the G-quadruplex [B7]-3-0 peroxidase DNAzyme [9]. Recently, a non-G-quadruplex Hem1 DNA aptamer was also selected through a capture-based selection method and shown to have peroxidase-like activity in binding with hemin [10].

These discovered DNAzymes hold significant potential for translational applications due to their low molecular weight, thermal stability, reversible denaturation, enhanced resistance to hydrolysis, and ease of chemical synthesis at a low cost. These features make them more attractive in biomedicine, diagnostics, DNA nanotechnology, and analytical chemistry applications [11]. However, reported peroxidase DNAzymes have prohibitively slow catalytic rates compared to their protein and RNA counterparts. Several approaches have been attempted to solve this including modifying DNA backbones, adding adenosine triphosphate to the reaction, linking adjacent adenines at the 3′-end of the G4-core sequence, using small molecular enhancers at higher concentrations, and affinity-based SELEX [9, 10, 12–16]. However, all these approaches have had challenges in the discovery of better DNAzymes. Evolutionary approaches which select for function-based peroxidase activity instead of simply binding could favour the selection of better DNAzyme variants.

**Figure 1.**
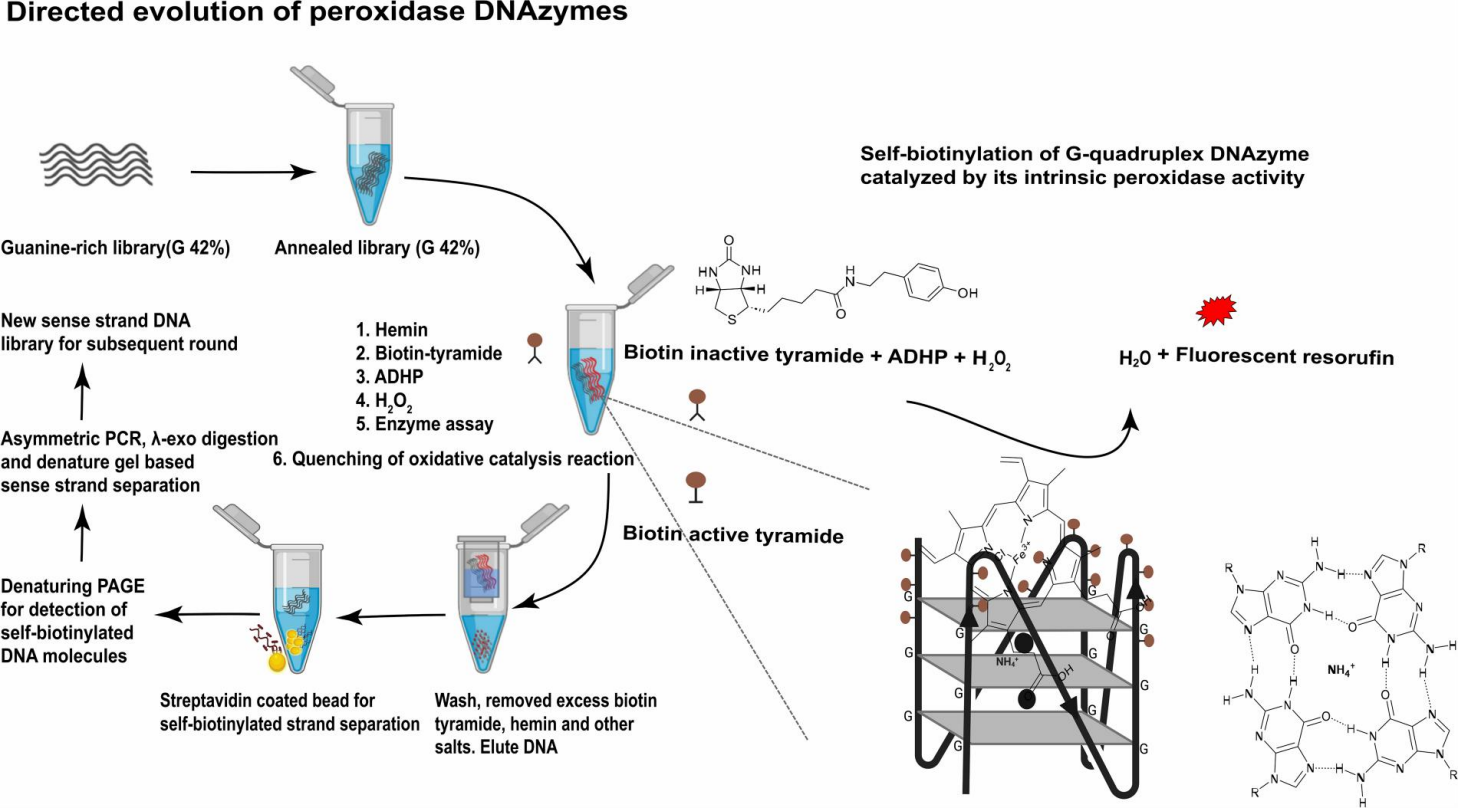
An overview of the functional-based directed evolution procedure for evolving peroxidase-mimicking DNAzymes. Method was adapted with permission from Bhuyan *et al*. [11], American Chemical Society

In this new protocol, we present the details of such a function-based directed molecular evolution procedure (Fig. 1). We captured short-lived tyramide radicals [17, 18] to select functional DNA molecules exhibiting peroxidase activity through a directed evolution experiment. This approach has facilitated the discovery of the mini self-biotinylated DNAzyme-X-3 (mSBDZ-X-3) from a guanine-rich DNA library [11]. In the presence of peroxidase substrates 10-acetyl-3,7-dihydroxyphenoxazine (ADHP), biotin-tyramide, and mild reactant H_2_O_2_, active G-quadruplex DNA/hemin triggers intrinsic peroxidase activity. This leads to an exponential increase of fluorescent signal in real-time and self-biotinylation reaction in the enzyme assay buffer. Subsequently, streptavidin-conjugated magnetic beads are used to sort the population of self-biotinylated DNAzymes from the DNA library after the quenching of the enzyme reaction. Once the enriched catalytic activity of DNA pools could be determined through fluorescent-based enzyme assay in a real-time and denaturing gel shift analysis, the selected pool was considered for cloning, sequencing, and downstream biochemical and biophysical characterization to deduce the active variant [11]. This protocol could also be used to discover peroxidase RNAzyme or DNAzyme from a guanine-rich natural or modified nucleotide library.

## Advantages over other methods

There are two major existing methods used for selecting DNAzymes. Li *et al*. developed an affinity-based *in-vitro* selection approach to discover DNA aptamers with high specificity and affinity for N-methyl mesoporphyrin IX (NMM). They attached NMM ligands to acrylic beads in a packed column while selecting DNA aptamers based on their target-recognition capability, binding selectivity, and affinity. After several rounds of classical SELEX and sequence characterisation, they identified DNA aptamers with sub-micromolar affinity for the NMM ligand. Serendipitously, two porphyrin-binding DNA aptamers were found which showed peroxidase activity with cofactor hemin in the presence of mild hydrogen peroxide [2, 3]. In 2012, Zhu and coworkers developed another affinity-based *in-vitro* selection approach targeting hemin ligands. They chemically linked cofactor hemin to the agarose beads' surface while selecting hemin-binding aptamers from DNA libraries containing varying percentages of guanine bases [9].

Such traditional selection strategies primarily rely on affinity instead of catalytic activity. In theory, fluorescence-based detection and capture of catalytic DNA during evolutionary selection rounds should be a more sensitive and robust approach as the selective pressure is catalysis instead of binding. Additionally, traditional selection strategies might take a longer time to discover DNAzymes than our approach, because ligands need to be packed in a flow column or covalently linked to the bead’s surface before the selection process begins.

In general, compared to affinity-based approaches, a function-based directed evolution method offers several advantages: (i) The strategy is solution-phase which is particularly important for selection for catalysis; (ii) the strategy continuously monitors specific enzyme activity based on fluorescence in real-time, thereby providing robust monitoring of DNA pool activity across selection rounds; and (3) the strategy uses self-labelling of DNAzymes with biotin-tyramide during selection, which is particularly sensitive for capture.

## Technical limitations

There are several advantages to using function-based directed evolution for selecting peroxidase DNAzyme, but there could be some technical limitations. In brief, this selection approach uses a guanine-rich library to enrich self-biotinylated DNA based on its catalytic activity. To determine the limitations of this present method, one could use this selection approach to evolve peroxidase DNAzyme from different DNA libraries including classical, different percentages of guanine, or modified nucleotide DNA libraries containing unnatural nucleotides in order to assess the enrichment efficiency. This system has not yet been tested for the selection of peroxidase RNAzymes. There is significant scope to extend this methodology to RNA and other types of nucleic acid chemistries.

## Potential applications

The strength of this evolution method lies in its ability to self-label and enrich DNAs based on peroxidase activity from the solution phase, makes it potentially useful for various applications. We anticipate that this laboratory evolution strategy will serve as a powerful tool for enriching peroxidase DNAzymes and discovering modified DNA peroxidase DNAzymes. This selection system could also be utilized to select for enantio-specific DNAzymes, and we anticipate that it may be compatible with isolating peroxidase RNAzymes from cell transcriptomics. Additionally, the approach could be applied to selecting catalytic activities using other porphyrin derivatives during the selection process.

## Overview of the procedure

The function-based directed evolution procedure is summarized in Fig. 1 and consists of the following key stages.

- A guanine-rich DNA library having 58 random nucleotide sequences with 42% guanine, flanked by constant primer binding sites, is used in evolutionary selection rounds as a positive, and one classical DNA library is used as a negative control.
- Equal amounts of DNA from each library are annealed, followed by adding cofactor hemin to the reaction mixture for binding to the structured DNA library. In this step, we use ∼0.5 nmol of DNA library as an initial molecule.
- Each reaction was mixed with peroxidase substrate Biotin-tyramide, followed by fluorogenic substrate ADHP in 15-min intervals.
- Time course enzyme assay was carried out after mixtures were transferred to a 96-well black plate, followed by auto injection of reactant H_2_O_2_.
- Quenching of the oxidative catalytic reaction of DNAzyme followed by purification of DNA pools.
- Enrichment of self-biotinylated DNAzyme pools by Streptavidin immobilized Dynabeads.
- Analysis of self-biotinylated DNAzyme pools.
- Sense strand DNA preparation of guanine-rich DNA and qualitative analysis for subsequent rounds.
- Cloning, sequence analysis, enzyme assay, and structure determination of identified DNAzyme variants.

## Experimental design

### Library and self-biotinylation reaction by intrinsic peroxidase activity

The nucleotide makeup within the library may impact structural diversity. We used a desalted guanine-rich DNA library with 42% guanine, flanked by fixed primer regions, to increase our chances of finding DNA sequences with peroxidase activity [11]. A classical DNA library is used as a negative control [11].

Guanine-rich DNA library:

5′-AAAGTTAGAAATGATGCTAAA-N58-TTTGACTCACATCAATGCAA-3′

Classic DNA library:

5′-AAAGTTAGAAATGATGCTAAA-N58-TTTGACTCACATCAATGCAA-3′

The self-biotinylation reaction is crucial in this functional-based directed evolution approach (Fig. 1). Cofactor hemin was initially allowed to bind with an annealed guanine-rich DNA library in an assay buffer containing alkali cations. Subsequently, peroxidase activities were determined in a 96-well black polystyrene microplate in the presence of fluorogenic substrate ADHP and biotin-tyramide in mild H_2_O_2_ through an auto-injector of Varioskan LUX multimode microplate reader. Substrate ADHP generates fluorescent signals under oxidation reaction, which determines the catalytic activity of the DNAzyme pool allowing real-time monitoring for that selection round. Biotin-tyramide promotes a degree of self-biotinylation response by conversion of inactive to active biotin-tyramide on the active sites of DNAzyme, which allows capture of self-biotinylated DNA molecules by MyOne Streptavidin T1 Dynabeads. Emission spectra were used to determine the initial velocity throughout selection rounds. We reduced the enzyme reaction time by 30–3 min through the ninth selection round. Quenching of enzyme assay on time is essential, which increases the chance of specific labelling of functional DNA molecules. Sodium pyruvate and Trolex were used to quench the reaction before DNA purification.

### Purification, enrichment, and evaluation of enriched self-biotinylated DNAzyme pools

After quenching the enzyme assay, DNA purification is carried out to remove interfering chemicals. Interfering chemicals may affect the enrichment efficiency of self-biotinylated DNA. We advise cleaning the assay product using the ssDNA/RNA Clean & Concentrator kit (Zymo Research), which should be included before capturing self-biotinylated DNAs.

To capture the self-biotinylated DNA molecules from the purified DNA pool, we used Dynabeads MyOne Streptavidin T1. To achieve the maximum chance of enriching efficient catalytic DNA molecules, we increased selective pressure by the following approaches: (i) reduced the enzyme reaction time by 30–3 min through nine selection rounds; (ii) reduced the DNA: tyramide ratio from the 4th to 9th rounds; (iii) reduced the DNA: hemin ratio from 1:3 to 1:2 from 8th to 9th rounds; (iv) reduced the capturing time of DNA pools from 45 to 15 min during the second to ninth selection rounds; and finally (v) increased washing stringency from 60 to 300 μl during selection rounds. We advise introducing selection pressure while performing the selection. This increases the chance of enriching efficient self-biotinylated functional DNA while eliminating weak or non-functional DNA sequences.

**Figure 2.**
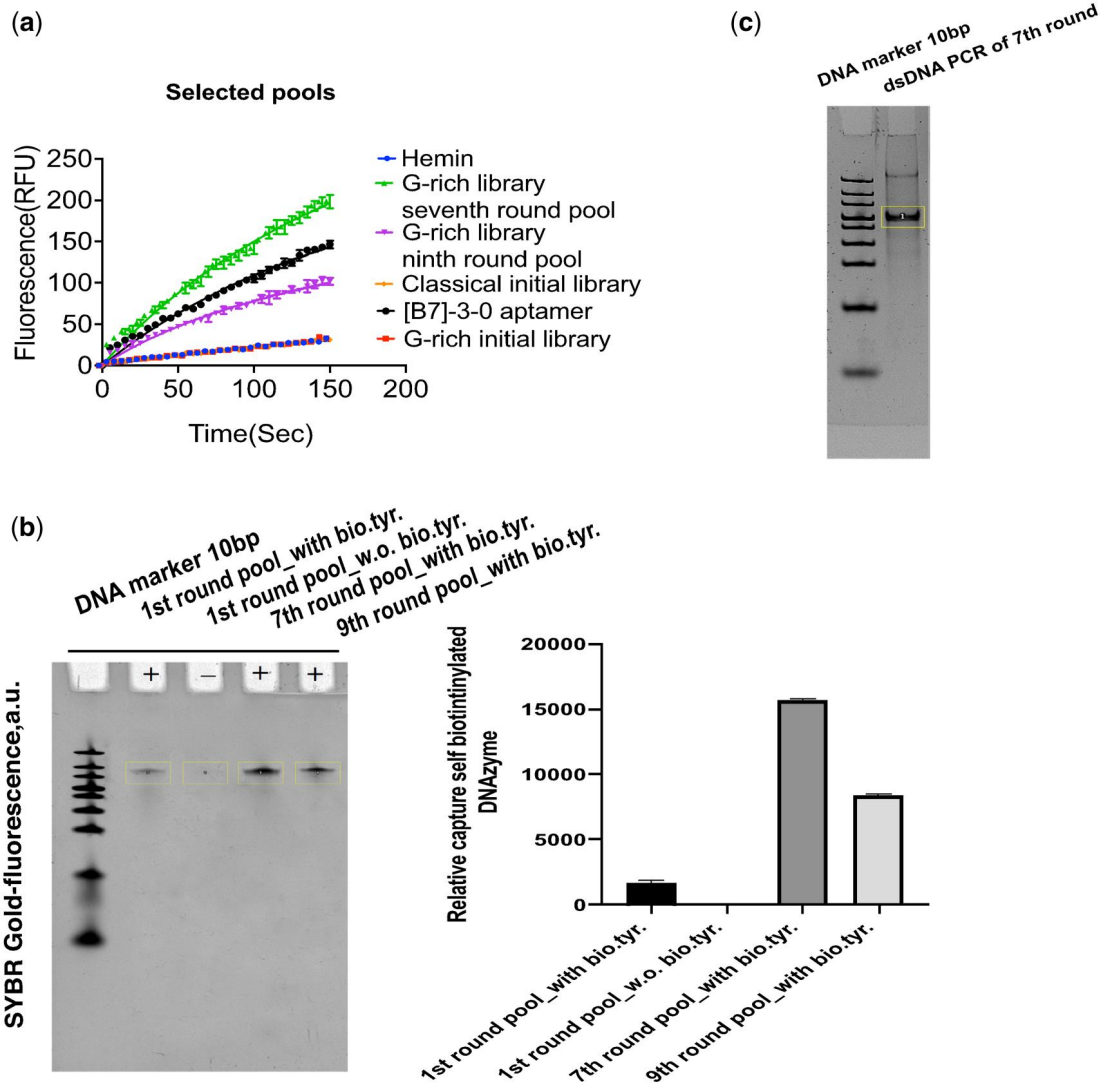
Characterization of selected DNAzyme pools. (**a**) Fluorescence-based enzyme assay measurements were used to determine the initial rate, V_0_ of the selected DNAzyme pools, where [B7]-3-0 aptamer and a classical DNA library used as positive and negative controls. Intrinsic peroxidase activity of the active G-quadruplex DNAzyme-heme (Fe^3+^) complexes facilitates the oxidation of fluorogenic substrate in the presence of mild oxidant H_2_O_2_. (**b**) Gel-shift and relative densitometric analysis show a functional enrichment of self-biotinylated DNAzyme pools. (**c**) Qualitative analysis of the seventh-round DNA pool PCR amplified product for cloning. The entire dataset was made and adapted with permission from Bhuyan *et al*. [11], American Chemical Society

Examining the efficiency of enrichment is essential as we enriched self-biotinylated DNA pools. To achieve this, we performed an enzyme assay to determine the velocity of enriched pools, and performed denaturing gel shift analysis of beads captured by self-biotinylated DNA molecules of selected DNA pools, measuring shifted DNA band intensity using gel densitometric analysis software, Image J. Both assay results showed the seventh-round DNA pool displayed higher velocity and intensity levels of shifted DNA band than other pools (Fig. 2a and b). Self-biotinylated DNA molecules were PCR amplified for the subsequent selection rounds.

### PCR amplification and processing of enriched bead-captured self-biotinylated DNA

Next, beads captured DNA molecules were PCR amplified through asymmetric PCR using the optimised ratio of 5’-phosphate-labelled reverse and standard forward primer. PCR amplification may increase the chance of selection by increasing the population of enriched molecules over selection rounds. Therefore, we used the following standard procedures for bead-based asymmetric PCR to generate the maximum chance of ssDNA molecules. The steps are: (i) optimized ratios of 5’-phosphate labelled reverse to forward primer along with the PCR cycles number were used for the maximum chance of amplification of ssDNAs within the pools; (ii) purification and concentration of PCR products were performed using ssDNA/RNA Clean & Concentrator kit (Zymo Research); followed by (iii) Optimized time of lambda exonuclease digestion is essential to get rid of antisense strand DNA in the mixture population of PCR product.

### Sense-strand DNA preparation and qualitative analysis

Asymmetric PCR does produce symmetric and asymmetric amplification of DNA molecules. Sense-strand DNAs are crucial for the directed evolution of peroxidase DNAzyme. To generate high-quality sense-strand DNAs, PCR products must be cleaned and concentrated before being processed through Lambda Exonuclease digestion and denature gel-based extraction. We cleaned and concentrated the PCR products to eliminate unused primers and other residuals using the ssDNA/RNA Clean & Concentrator kit. Concentrated DNA products were mixed with 1× digestion buffer and 5U Lambda Exonuclease enzyme to eliminate phosphate-labelled anti-sense-strand DNA. After 20 min of digestion at 37°C under shaking (120 rpm), products were treated with EDTA and heated at 80°C to terminate the reaction.

Digested products were mixed with 1× DNA loading buffer and passed through the denaturing preparative gel electrophoresis (12%) to recover homogeneous sense-strand DNA. The synthetic ssDNA library pool and asymmetric PCR product (internal controls) were passed through different lanes in the same extraction gel in parallel to confirm the correct size of ssDNA extracted from the PAGE. The desired DNA band of interest was detected after staining the gel for 30 min with SYBR-Gold. A sterile knife was used to remove a gel slice from the stained gel under the SYBR-gold filter. The gel slice was cut into smaller fragments to increase the yield of ssDNA release from urea-PAGE to the extraction buffer. DNA extraction buffer was added to a polypropylene tube containing a gel slice and incubated at 37°C, 180 rpm for 12–16 h to release most of the ssDNA from the gel slice, followed by centrifugation for 1 min at 13 000 rcf. To increase the recovery, procedures were repeated by adding one volume of DNA extraction buffer to the gel slice containing tube and vortexing for 5 min before centrifugation. Extracted supernatant was mixed with twice the volume of the binding buffer of ssDNA/RNA Clean & Concentrator kit and processed for cleaned, concentrated, and recovered in ultra-pure DNAase/RNAase-Free distilled water for downstream qualitative and quantitative analysis before use in the subsequent selection round.

To confirm the quality of sense-strand DNA recovered from the denaturing PAGE, we used a Cy5 labelled reverse primer molecular probe to determine the quality of sense-strand DNA in a hybridisation mechanism by detecting through native PAGE under Cy-5 filter through selection rounds (Fig. S1). We used internal controls (G-rich library hybrid with Cy5-probe, G-rich-DNA library without Cy-5 probe, asymmetric PCR with Cy-5 probe, and PAGE-purified sense strand DNA with Cy5 probe) during gel analysis through Image J analysis software. We also evaluated the quantity of DNA using DNA nanodrop before passing through the subsequent selection rounds.

### Cloning and downstream analysis of evolved DNAzyme

It is necessary to visualize the enriched sequences of self-biotinylated DNA molecules. To do so, we cloned the seventh-round pool as it exhibited the highest peroxidase and self-biotinylation activity. The seventh-round DNA pool was PCR amplified using the equal ratio of forward and reverse primer without 5’-phosphate modified using high-fidelity DNA polymerase enzyme (Fig. 2c). The amplified product was further extracted through PAGE gel and cleaned and concentrated using Wizard SV gel and PCR Clean-up system (Promega). The extracted DNA product was ligated further into the PCR-Blunt II-TOPO vector before transforming into TOP10 competent cells (Life technology). Thirty colonies were randomly picked and employed for Sanger sequencing. Subsequently, DNA sequences were analysed using the Multiline tool for enrichment analysis and the QGRS mapper tool to analyse GQ motifs and distribution patterns among sequences (Fig. S2) [11]. Sequence analysis deduces five dominant DNA sequences (Fig. S2) [11].

**Figure 3.**
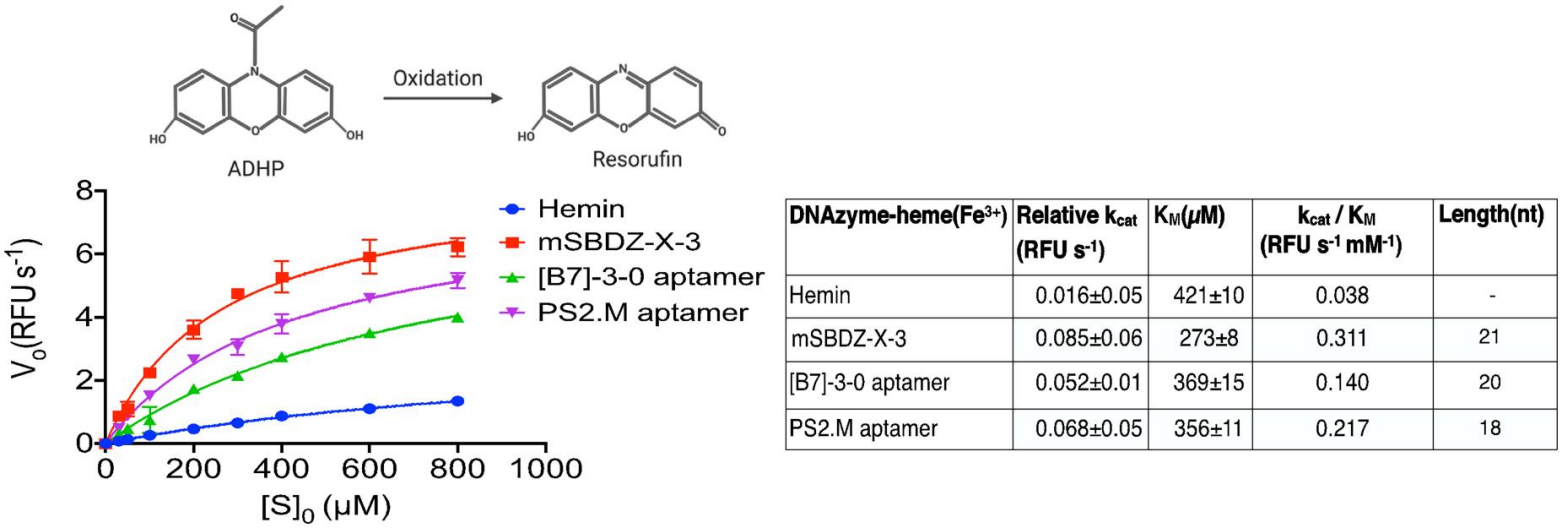
Kinetics study of the evolved and reported DNAzyme variants. Michaelis−Menten kinetics of the peroxidation reaction catalysed by the DNAzyme−heme (Fe^3+^) in the presence of H_2_O_2_. Enzyme-specific reaction oxidises the peroxidase substrate ADHP to the fluorescent oxidized product, 7-hydroxyphenoxazin-3-one (resorufin). The table highlights the kinetics parameters deduced from the best-fit model of four test samples. The dataset was made and adapted with permission from Bhuyan *et al*. [11], American Chemical Society

**Figure 4.**
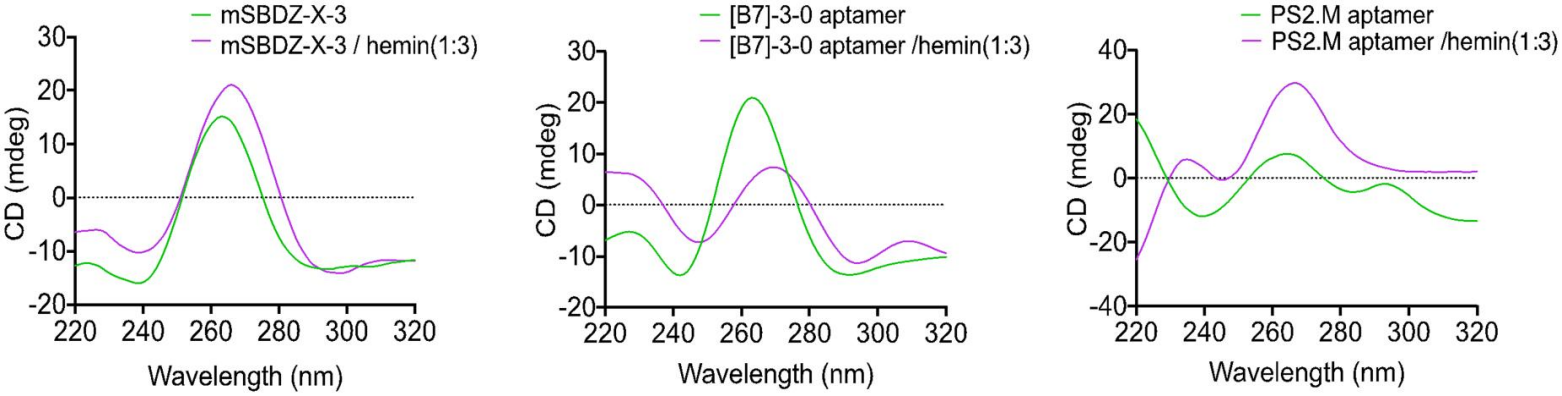
Structural analysis of DNAzyme variants. Circular dichroism (CD) of mSBDZ-X-3, [B7]-3-0 aptamer, and PS2.M aptamer spectra show in the presence and absence of cofactor hemin. The lavender line represents the change in spectra after binding with cofactor hemin. The dataset was made and adapted with permission from Bhuyan *et al*. [11], American Chemical Society

Further, detailed biochemical and biophysical characterisation deduced the best peroxidase DNAzyme variant, mini self-biotinylated DNAzyme-X-3 (mSBDZ-X-3) [11]. We additionally performed an enzyme assay to pursue kinetic at an optimised molar ratio of cofactor hemin in a time-dependent manner in varying H_2_O_2_ concentrations. From the enzyme kinetics, we determined K_M_ and V_max_ using the Michaelis–Menten equation and efficiency constant using the lim Ef =K_cat_/K_M_ equation (Fig. 3). Kinetics results showed the mSBDZ-X-3 holds promising enzyme kinetic properties concerning reported variants ([B7]-3-0 aptamer, PS2.M aptamer, and EAD2). The CD spectroscopy results further revealed the mSBDZ-X-3 is a parallel G-quadruplex structure (Fig. 4).

## Materials and methods

### Reagents

- This protocol used standard desalted ssDNA classical, 42% G-rich library and HPLC grade primers from IDT (see Experimental design section for more details about sequence information)
- Dual HPLC grade 5’-phosphate labelled reverse primer used for asymmetric PCR amplification and lambda exonuclease treatment for readout anti-seance strand DNA after PCR
- HPLC grade 5’-Cy5 labelled reverse primer-probe used for qualitative analysis of recovered sense strand DNAs from denatured PAGE
- 0.2 ml, thin wall, clear eight-strip PCR tubes and microfuge tubes (Axygen)
- Microfuge tubes, 1.5 ml and conical tubes, 15 ml and 50 ml (Axygen and Falcon)
- UltraPure 1M Tris–HCl pH 7.5 (Invitrogen)
- Water (UltraPure™ DNase/RNase-Free Distilled Water; Thermo Fisher Scientific, cat. no. 10977015)
- NH_4_Cl (Sigma-Aldrich)
- KCl (Sigma-Aldrich)
- NaCl (Sigma-Aldrich)
- Triton X-100 (usb.)
- Hemin (Sigma Aldrich)
- Biotinyl tyramide HPLC grade (Sigma-Aldrich)
- 10-Acetyl-3,7-dihydroxyphenoxazine (ADHP) (Cayman Chemical)
- 9.8 M H_2_O_2_ (BDH-prologue)
- Sodium pyruvate (Thermo Scientific)
- Trolex (Sigma-Aldrich)
- ssDNA/RNA Clean & Concentrator kit (Zymo Research)
- Dynabeads MyOne Streptavidin T1 (Invitrogen, Life Technologies)
- PB buffer (Qiagen)
- dNTPs (Invitrogen, Life Technologies)
- PWO super yield DNA polymerase enzyme (Roche)
- Ultra-Low Range DNA ladder (Invitrogen)
- 10U/uL Lambda Exonuclease and digestion buffer (Thermo Fisher Scientific)
- EDTA (Invitrogen, Life Technologies)
- Urea (Sigma-Aldrich)
- Native PAGE (Bio-Rad 30% acrylamide and bis-acrylamide solution, 29:1 used in the preparation)
- Denature PAGE (Bio-Rad 30% acrylamide and bis-acrylamide solution, 29:1 with urea from Sigma used in the preparation)
- SYBR-Gold (Invitrogen, Life Technologies).
- Formamide (Sigma-Aldrich)
- Wizard SV gel and PCR Clean-up system (Promega)
- Zero Blunt TOPO PCR Cloning Kit (Life technology)
- Top10 chemically competent *E. coli* (Life technology)
- 15 ml polypropylene tube (SPL Life Science)
- 30% Acrylamide/Bis-acrylamide solution (29:1) (Bio-Rad)
- N, N-Dimethylformamide (Sigma-Aldrich)
- Dimethyl sulfoxide (Sigma-Aldrich)
- TEMED (USB)
- APS (Sigma-Aldrich)
- DNA gel loading dye 5× (QIAGEN)
- Plasmid isolation Kit (QIAGEN QIApeep Spin miniprep kit)

### Equipment

- Weighing scale (Sartorius)
- Heat block (Labnet)
- Thermal cycler (Applied Biosystems, Life technology)
- 96-well black polystyrene microplate (Perkin Elmer)
- Orbital shaker (Genie)
- HulaMixer (Invitrogen)
- Dynal MPC-S Dynabeads Magnetic Particle Concentrator (Applied Biosystems)
- Incubator Shaker (MaxQ 4450, Thermo Scientific)
- Microcentrifuge-ScanSpeed Mini (Gene speed X1)
- Benchmark myFuge Mini Clear lid (European 2-prong plug)
- Vortex 2(Genie)
- Varioskan LUX multimode microplate reader (Thermo Scientific)
- Water Bath (Boeco Germany)
- DNA Electrophoresis Equipment (Bio-Rad)
- ChemiDoc Imaging System (Bio-Rad)
- UV Transilluminator (FOTODYNE INC)
- Disposable surgical blade (PARAGON)
- NanoDrop 100 spectrophotometer (Thermo Scientific)
- CD spectrophotometer (JASCO, J-1500)

### Software

- Excel 2019
- GraphPad Prism Software 8.0
- Image J
- Alignment server (Multalin)
- QGRS mapper server

### Reagent setup

- Buffers and solutions
  - Stock enzyme assay buffer 10× (pH 7.57): 500 mM Tris–HCl pH 7.5, 1.5M NH_4_Cl, 0.3% Triton X-100 prepared in ultrapure water and stored at room temperature.
  - Hemin stock: ∼1.533 mM, prepared in dimethylformamide (DMF) and stored at -20°C. **NOTE:** Hemin is light sensitive.
  - Biotin tyramide (HPLC grade) stock ∼5.00 and 100 mM 10-acetyl-3,7-dihydroxyphenoxazine (ADHP) stock prepared in dimethyl sulfoxide (DMSO) and stored at −20°C. **NOTE:** ADHP is light-sensitive.
  - Before the enzyme assay, 3 μM H_2_O_2_ was prepared immediately from the stock (9.8 M) in MQ autoclave water.
  - Neutralisation buffer for enzyme assay: ∼60 mM of sodium pyruvate and ∼5 mM of Trolex prepared in PBS.
  - Nucleic acids Dyna beads MyOne Streptavidin T1 binding (2×) buffer: 10 mM Tris–HCl pH 7.5, 1 mM EDTA, 2 M NaCl. For washing, dilute 2× binding buffer to 1× using an equal volume of Ultrapure DNase/RNase-Free Distilled Water.
  - PCR set up for optimizing annealing temperature and PCR cycle number to optimize best amplification output.
  - 10× Lambda Exonuclease digestion buffer dilute to 1× in ultrapure water for lambda exonuclease treatment for readout a 5’-phosphate labelled anti-seance strand DNA of PCR product.
  - 12% Urea-PAGE: Mix 14.4 g Ultra-pure Urea (8 M) with 40% Acrylamide/Bis-acrylamide solution (29:1) in nuclease-free water in addition to 30% APS, 1× Ultra-Pure TBE buffer and TEMED.
  - 12% Native-PAGE: 30% Acrylamide/Bis-acrylamide solution (29:1) in nuclease-free water in addition to 10% APS, 1× Ultra-Pure TBE buffer and TEMED.
  - 1× PAGE running buffer prepared in MQ water by diluting 10× Ultra-pure 10× TBE Buffer.
  - PAGE staining buffer prepared in Ultra-Pure 1× TBE buffer by diluting 1× SYBR Gold from the stock.
  - 95% Formamide prepared and stored at 4°C.
  - 1× DNA loading buffer for denaturation PAGE (90% formamide, 0.5% EDTA, 0.1% bromphenol blue and 0.1% Xylene cyanol in water).
  - DNA extraction buffer for denature and native PAGE: 10 mM Tris–HCl (pH 7.5), 210 mM NaCl, 1 mM EDTA in Ultrapure DNase/RNase-Free Distilled Water
- Primers and Library
  - Prepare library and all primer stock in Ultra-pure DNase/RNase-Free Distilled Water before use.

### Equipment setup

- Pre-set the Varioskan LUX multimode microplate reader (Thermo Scientific) plate layout and protocol (i.e. H_2_O_2_ dispense volume (10 μl) at medium dispensing speed; Shake parameters (duration 05 ss at 600 spm speed with diameter 1 mm and end with ON time shaking mode; Fluorometric parameters λex = 558 nm, λem = 585 nm, excitation bandwidth (5nm), dynamic range (Autorange), measurement time with optics (top) for ADHP; kinetic Loop readings with interval of 01 ss; Incubate temperature parameter set at 25°C before carried out enzyme assay by injecting H_2_O_2_ through an auto-injector.
- Pre-run the denature preparative PAGE at 37°C at 100 volts at least 30 min before the sample loading.
- Pre-set the emission spectrum of the oxidised fluorogenic substrate (ADHP) at λex = 558 nm, λem =585 nm.

### Procedure

**A. DNA library preparation and enzyme assay for selection.**

1. Add ∼0.5 nmol of guanine-rich DNA library to 160 μl of reaction mixture containing nuclease-free water and 1× enzyme assay buffer in a micro-centrifuge tube, mix and denature at 95°C for 10 min and slowly cool the mixture to 25°C over 1 h. **NOTE**: Prepare ∼ 0.5 nmol of guanine-rich DNA library in a separate reaction as a negative control without biotin tyramide. Additionally, take ∼0.5 nmol of classical DNA library as a negative control in 160 μl of reaction mixture containing nuclease-free water and 1× enzyme assay buffer in a micro-centrifuge tube, mix and anneal as Step 1. The self-biotinylation reaction by intrinsic peroxidase activity.
2. Add 10 μl of ∼300 μM hemin to the individual mixture from Steps 1, mix and incubate at RT for 30 min with shaking **(Avoid light). NOTE:** Freshly prepare the hemin dilution stock in DMF before use.
3. Add 10μl of ∼250 μM biotin-tyramide to reaction tubes, mix and again incubate at RT for 15 min **(Avoid light). NOTE:** Freshly prepare the biotin-tyramide dilution stock in DMSO before use.
4. Prepare and add 10 μl of ∼25 mM ADHP to the individual mixture from Step 3, mix well and transfer to a 96-well black polystyrene microplate. **(Avoid light). NOTE:** Freshly prepare the ADHP dilution stock in DMSO before use.
5. Transfer the 96-well plate to the pre-set Varioskan LUX multimode microplate reader.
6. Inject 10 μl of ∼6 mM H_2_O_2_ to the wells of the plate *via* an auto-injector to start the enzyme reaction at 25°C. **NOTE:** Pre-calibrate the auto-injector of the microplate reader before actual injection and prepare the H_2_O_2_ dilution stock in UltraPure™ DNase/RNase-Free Distilled Water right before use.
7. Continuously record the ADHP fluorescence emission spectrum at λ_ex_ = 558 nm, λ_em_ = 585 nm up to 30 min in the first selection round by the fluorometric mode of the Varioskan LUX multimode microplate reader. **NOTE**: We used 5 min record for both the second and third rounds and 3 min for the fourth to ninth rounds of the selection. Enzyme reaction time can be varied.
8. Quench the enzyme reaction by adding prechilled ∼60 mM of sodium pyruvate and ∼5 mM of Trolex mixture into the wells and mix and hold for 5 min.
9. Analyse the data from Step 7 and determine the initial velocity using the equation V0 = change in RFU/unit time (0–150 s) in Excel and GraphPad Prism Software.
**B. Capture of self-biotinylated DNA molecules and sample preparation for asymmetric PCR.**
10. Purify ssDNA using ssDNA/RNA Clean & Concentration Kit (**Zymo Research)** after quenching the enzyme assay from Step 8. **NOTE:** Purification should be performed immediately after quenching of the enzyme assay. More prolonged incubation without purification might lead to non-specific labelling. Determine the DNA concentration using a nano-drop or Qubit assay and store it in an elution buffer (Qiagen) at 4°C for subsequent downstream experiments.
11. Pre-wash ∼400 μg of Dynabeads MyOne Streptavidin T1 using the Invitrogen, Life Technologies guideline. **NOTE:** We used ∼200 μg of Dynabeads MyOne Streptavidin T1 from the second to ninth selection rounds.
12. Incubate wash Dynabeads with purified ssDNA pool from Step 10 for 45 min at RT in a gentle rotation using HulaMixer to capture self-biotinylated DNA molecules from initial selection rounds. **NOTE:** We used 45 min of capturing time in the 1st to 3rd rounds, 25 min in the 4th to 6th rounds, and a final 15-min duration in the 7th to 9th rounds. Capturing time can be varied through selection rounds.
13. Post-wash Dynabeads 3–6 times with 1× B&W Buffer (Invitrogen, Life Technologies) after capturing self-biotinylated DNA molecules. **NOTE:** We used ∼60 μl of washing volume in the 1st to 3rd rounds, ∼100 μl in the 4th to 6th rounds, and a final ∼300 μl washing volume during the 7th to 9th rounds of selection. Washing volume can be varied.
14. Resuspend the wash capture Dynabeads in EB buffer (Qiagen) for asymmetric PCR and evaluation of the degree of self-biotinylation activity using denaturing PAGE. **NOTE:** Capture DNA should be stored at 4°C for a few weeks.
15. Dilute 90% Dynabeads captured DNA molecules in 1× PWO Super Yield PCR buffer mixed with nuclease-free water for asymmetric PCR.
16. Add HPLC purified 0.5 pmol of the forward primer and an optimized 0.15 pmol of dual HPLC purified reverse primer link with a 5’-phosphate along with 0.5 mM dNTPs and 2.5 U PWO super yield DNA polymerase enzyme.
17. Mix the PCR mixture carefully by pipetting and amplify the DNA pool for 25–28 cycles after transferring the samples to the thermocycler (Applied Biosystems, Life technology) using a cycling program: 95°C for 2 min, 95°C for 15 s, 60°C for 30 s, 72°C for 45 s, 72°C for 2 min, and final hold at 4°C for infinity. **NOTE:** PCR cycle number should be optimised during selection rounds.
18. Load the amplified PCR product onto the ssDNA/RNA Clean & Concentrator kit to recover DNA from the PCR buffer and residual primers.
19. Monitor the PCR amplification on a 12% native TBE-PAGE. For this, load 1–2 μl of PCR product mixed with 1× DNA loading buffer and run until the DNA ladder is well separated.
20. Carefully transfer the gel into gel staining 40 ml TBE buffer mixed with SYBR-Gold stain. Leave the gel for 15–30 min on a slow-moving shaker in the dark.
21. Scan the gel under the SYBR-Gold filter and determine the quality of the amplification product. **NOTE:** Always compare the positive PCR amplification with negative control for contamination.
**C. Lambda Exonuclease digestion.**
22. Mix 150 μl of PCR clean product with 20 μl of 10× Lambda Exonuclease Reaction Buffer and 5 μl of (10 U/μl) of Lambda exonuclease (from Step 18, after cleaning up asymmetric PCR product).
23. Incubate reaction for digestion at 37°C, 200 rpm for 20 min. Digestion time can be varied.
24. Terminate enzyme reaction using EDTA followed by heating at 80°C using a heat block for 10 min.
**D. Denaturing PAGE-based separation of sense-strand DNA.**
25. Prepare 12% denaturing PAGE. Dissolve 14.4 g ultra-pure Urea (8 M) in nuclease-free water and 1× TBE buffer (Ultra-Pure™ DNase/RNase-Free Distilled Water; Thermo Fisher Scientific) in a 15 ml polypropylene tube by applying gentle agitation at 37°C water bath with brief vortex. Add 40% Acrylamide/Bis-acrylamide solution (29:1) along with 30% APS and TEMED 30% (w/v) to dissolve urea solution. Make up the desired reaction volume with nuclease-free distilled water.
26. Assemble the 1.5 mm glass plates (Bio-Rad) and pour the dissolved solution into the gel-casting chamber using a serological pipet for polymerisation. Use a preparative wide spacer during the casting of gel.
27. Pre-run the gel for 20 to 30 min in 100 Volts using 1× TBE running buffer at 37°C using Bio-Rad-power-PAC 200.
28. Mixed digested DNA sample from Step 24 with 1× DNA loading buffer (90% formamide, 0.5% EDTA, 0.1% bromophenol blue).
29. Incubate sample for 5–10 min at 95°C for heat denaturation.
30. Rinse the fused wells thoroughly using 1× TBE buffer.
31. Load the DNA samples carefully into the loading wells right after heat denaturation, run the gel at 80 V for 30 min, and later increase the voltage to 100 V.
32. Observe the migration trends of the DNA ladder and dye in front of the end of the gel.
33. Carefully transfer the gel into gel staining TBE buffer mixed with SYBR-Gold stain. Leave the gel for 15–30 min in a slow-moving shaker in the dark.
34. Scan the gel under the SYBR-Gold filter and determine the quality of the ssDNA pool in the final PCR-digested product. **NOTE:** Perform the qualitative analysis of ssDNA according to the molecular size by comparing it to the positive controls (synthetic ssDNA library and initial asymmetric PCR product).
**E. PAGE extraction of ssDNA.**
35. Excise the DNA fragment of interest from denatured PAGE gel according to the molecular size, then cut it into smaller fragments using a sterile blade.
36. Transfer gel fragments to a weighed 15 ml polypropylene tube.
37. Add two volumes of PAGE DNA gel extraction buffer per mg of gel slice.
38. Close the tube and incubate at 37°C or RT at 180 rpm for 12–16 h.
39. Centrifuge the sample at 12–13K rcf for 1 min at RT.
40. Transfer the supernatant to a fresh 15 ml polypropylene tube.
41. Add one volume of gel elution buffer to the tube as gel weight.
42. Vortex for 5 min, centrifuge again at 12–13K rcf for 1 min at RT and combine the supernatants.
43. Mix the supernatant content DNA volume with twice the volume of binding buffer and follow the remaining stapes for extraction of ssDNA as per the Zymo Research ssDNA/RNA Clean & Concentrator kit.
44. Recover ssDNA (sense strand DNA) and store in Ultrapure DNase/RNase-Free Distilled water, measure concentration using Nano-Drop and store at −20°C until qualitative confirmation using native PAGE and use for subsequent selection rounds. **NOTE:** Samples can be stored at −20°C at this stage.
**F. Native PAGE-based qualitative detection of sense-strand DNA using Cy5 labelled probe.**
45. Mix recovered DNA from Step 44 with a Cy5-tagged Reverse primer probe (0.5 pmol) for hybridisation at RT or 25°C for 10 min in the dark.
46. Prepare and pre-run the 12% native PAGE in 100 V at RT for 10–15 min.
47. Load the mixed DNA samples into the gel wells after mixing with 1× DNA loading dye. Run the gel at 80 volts at RT using 1× TBE running buffer without light at RT.
48. After electrophoresis, DNA bands were visualised by scanning the gel in Cy5-filter (695/55) using a Bio-Rad Imaging system.
49. Again, the same gel stain with SYBR-Gold for 15–30 min was used, and DNA bands were detected under the SYBR-Gold filter (530/28) using the same Bio-Rad instrument imaging system.
50. To detect the sense-strand DNA over dsDNA in the extracted sample, compare the gel images of Cy5 exposure with SYBR-Gold exposure filters. Use internal controls (G-rich library with Cy5-R.primer, G-rich library, asymmetric PCR with Cy5-R.primer) for comparison.
**G. Second to the ninth round of selection.**
51. A fixed concentration of purified DNA from each pool (∼ 262.39 nM) from step 44, along with freshly prepared hemin (∼787.17 nM), biotin-tyramide (∼1.311 μM), ADHP (∼131.19 μM), and H_2_O_2_ (∼300 μM) were used in the second to seventh rounds of selections. We used a lower molar ratio of hemin (∼524.78 nM) in the eighth and ninth rounds. **NOTE:** Concentration of the reagents can be varied.
52. Experiment, enzyme assay, and analysis were performed the same way as Steps 1 to 10, excluding concentration of assay reagents and time. **NOTE**: 5 min record for both the second and third rounds and 3 min for the fourth to ninth rounds of the selection.
53. DNA purification after quenching of enzyme assay followed by capturing self-biotinylated DNA molecules to downstream extraction of ssDNA through PAGE and post qualitative analysis of ssDNA molecules from second to ninth rounds selection was performed as similar to Steps 11–50.
**H. Selected DNA pools for comparative enzyme activity.**
54. For comparative analysis, ∼100 nM of DNA from each selected pool and controls, ∼300 nM of hemin, ∼50 mM of ADHP, and ∼300 mM of H_2_O_2_ were used for 3 min enzyme assay excluding biotin-tyramide in the assay.
55. Experiment, enzyme assay and analysis were performed as in Steps 1–9, excluding the concentration of assay reagents.
**I. Determination of levels of self-biotinylation activity.**
56. Use 20 μg of beads captured self-biotinylated DNA pools from step 14 (initial round of guanine-rich DNA pool with and without biotin-tyramide) and step 55 (seventh and ninth rounds).
57. Add 1× loading buffer (90% formamide, 0.5% EDTA, 0.1% bromophenol blue) and heat samples at 95°C for 5 min.
58. Place the magnate immediately after denaturation and transfer all DNA-containing supernatants to fresh PCR tubes.
59. Pass DNA samples through precast 12% UREA-PAGE using TBE buffer and run at 100 volts. **NOTE**: Prepare 12% UREA-PAGE as per the mentioned recipe in Step 25. Pre-run the UREA-PAGE at 37°C for 20–30 min at 100 volts using Bio-Rad-power-PAC 200 before loading the samples. Use 1× TBE running buffer for electrophoresis.
60. Run the gel initially at 80 V for 30 min and then change to a constant 100 V until the dye front has reached the end of the gel.
61. Transfer the gel carefully to a cover or dark box containing 1× TBE buffer and SYBR Gold.
62. Leave the gel for 20–30 min of staining with SYBR-Gold by placing the box in a slow-moving orbital shaker in the dark.
63. Scan the gel under the SYBR-Gold filter (530/28) using the Bio-Rad(ChemiDocTMMP) Imaging system to identify the shifted DNA bands of interest.
64. Use the Image J analysis software to perform the qualitative densitometric analysis of shifted DNA bands among controls based on the SYBR-Gold fluorescence emission intensity. **NOTE**: Replicates should be used for gel analysis.
**J. Cloning and sequencing of the enriched DNA pool.**
65. For downstream analysis, 5% beads captured in the seventh round enriched DNA pool from step 53 diluted in 1× PWO Super Yield PCR buffer mixed with nuclease-free water for symmetric PCR.
66. Add HPLC purified 0.5 pmol of the forward and reverse primer along with 0.5 mM dNTPs and 2.5 U PWO super yield DNA polymerase enzyme for 100 μl of PCR reaction.
67. Prepare PCR mixture carefully by pipette and amplify the DNA pool for 30 cycles after transferring the samples to the thermocycler (Applied Biosystems, Life technology) using a cycling program: 95°C for 2 min, 95°C for 15 s, 60°C for 30 s, 72°C for 45 s, 72°C for 2 min, and final hold at 4°C for infinity. **NOTE**: Prepare a negative control PCR without a template to check for contaminations.
68. Monitor the target amplification at a 12% PAGE. For this load, 1–2 μl of each positive and negative PCR product was mixed with 1× DNA loading buffer and passed through the gel using TBE buffer. Run the gel until the DNA ladder is well separated. **NOTE**: Compare the target PCR product with negative control to check for contaminations after PCR.
69. The rest of the dsDNA PCR product from Step 67 was cleaned through the Wizard^®^ SV Gel and PCR Clean-Up System (Promega).
70. The cleaned PCR product was mixed with 1× DNA loading buffer and separated in 12% native PAGE. dsDNA extraction was performed as Steps 35–42.
71. Mix the supernatant content of dsDNA with binding buffer and follow the remaining stapes for extraction of dsDNA as per the Wizard^®^ SV Gel and PCR Clean-Up System (Promega). **NOTE**: The dsDNA purified product can be stored at −20°C for future use.
72. Mix PCR extracted of 3 μl from Step 71 with PCR-Blunt PCR-Blunt II-TOPO vector reagents (Life technology cloning kit) and incubate for 30 min at room temperature.
73. Place the reaction on ice for transformation.
74. Add 2–3 μl of ligated product to a vial of One Shot chemically competent TOP 10 (Life technology) 14 cells on ice for transformation.
75. Incubate on ice for 15–30 min, followed by heat shock for 30 s at 42°C water bath without shaking.
76. Immediately transfer the tube to ice and leave for 10 min.
77. Add 250 μl of S.O.C. medium to the tube at room temperature.
78. Incubate the tube at 37°C for 1 h at 120 rpm.
79. Spread different volumes (30–130 μl) into Kanamycin prewarmed selective plate and incubate plates overnight at 37°C. **NOTE**: Follow the Life technology cloning kit guidelines for better transformation efficiency.
80. Make master plates of bacteria colonies.
81. Pick 40 random colonies using a tip for colony PCR by picking colonies into 1.5 ml microfuge tubes having 10 μl of DNAase/RNAase-Free distilled water.
82. Incubate tubes at 95°C for 10 min for release of DNA.
83. Centrifuge the samples at 13K rcf for 5 min at RT.
84. Prepare 15 μl of a PCR reaction using 3 μl of the supernatant from Step 83 as a template DNA. **NOTE**: Always prepare negative colony PCR without a template to check for PCR contaminations.
85. Add 0.5 pmol of the forward and reverse primer along with 0.5 mM dNTPs and 2.5 U PWO super yield DNA polymerase enzyme to the PCR master mixture. Mix samples properly and transfer to thermocycler for 25 cycles of amplification using the following program: 95°C for 2 min, 95°C for 15 s, 60°C for 30 s, 72°C for 45 s, 72°C for 2 min, and final hold at 4°C for infinity.
86. Monitor the positive PCR amplification product on 12% native PAGE by loading 4–5 μl of PCR product mixed with 1× DNA loading dye.
87. Run the gel until the DNA marker is well separated. **NOTE**: Always run negative colony PCR for comparison with a positive amplified product of interest.
88. Prepare 10 ml overnight LB broth cultures of 30 random positive colonies having Kanamycin selection pressor in 15 ml polypropylene tubes at 37°C and 120 rpm.
89. Harvest plasmid from broth cultures using a Qiagen mini purification kit according to the manufacturer’s instructions.
90. Aliquot 50–100 ng plasmid DNA for Sanger sequencing using a universal pCR-Blunt II-TOPO vector internal primer.
91. Identify evolved random sequences from raw sequences using target-specific forward and reverse primers.
92. Perform multiple sequence alignment (MSA) using an online alignment server such as Multalin [19] to know the copy number, followed by a QGRS mapper server to analyse GQ motifs and distribution patterns within sequences [20].
93. Select five dominant full-length DNA sequences in Table S2 [11] without primer regions for further characterisation.
**K. Enzyme assay, kinetics and structural analysis of mSBDZ-X-3.**
94. Synthesise selected five sequences from step 93 and test them all for peroxidase activity using an enzyme assay buffer (50mM Tris-HCl, pH 7.5) with different monovalent cations, ie NH4Cl, NH4Cl/KCl, and KCl—experimental set-up as described in the method section [11].
95. Select the best sequence from step 94 and truncate into three residues Table S2 [11] to obtain the minimal catalytic core region based on enzyme activity and CD spectral analysis—experimental set-up described in the method section [11].
96. Obtain the catalytic core region from step 95 and further improve the enzyme activity by a terminal and internal loop nucleotide modification within the core nucleotide sequence and full-length DNAzyme sequence using addition and substitution of nucleotides—experimental set-up described in the method section [11].
97. Perform comparative biophysical characterisation of the evolved DNAzyme variants (SBDZ-X-5’-5Δ-3’-6Δ-A3, mini SBDZ-X-3-1, and mSBDZ-X-3) with reported aptamers (PS2.M aptamer, [B7]-3-0 aptamer, EAD2) using G4- ligands (ThT and NMM) fluorescent turn on assay, UV-vis, CD spectral analysis in bind with G4-ligands in the presence of monovalent and divalent cations (Zn2+, Mg2+, Ni2+), enzyme assays in different peroxidase substrates (ADHP, ABTS, Luminol) and temperature—experimental set-up as described in the method section [11].
98. Select mSBDZ-X-3 from step 97 and perform enzyme assay in varying ratios of cofactor hemin to obtain the correct balance for best enzyme performance—experimental set-up as described in the method section [11].
99. Denature 100 nM of selected DNAzyme variants (mSBDZ-X-3, PS2.M aptamer, and [B7]-3-0 aptamer) in a 1× enzyme assay buffer (50 mM Tris- HCl pH 7.5, 150 mM NH4Cl, and 0.03% Triton X-100) at 95°C for 10 min followed slowly cooled to 25°C for over 1 hr. for enzyme kinetic analysis.
100. Annealed DNA mixtures were allowed to bind with 300 nM of cofactor hemin (freshly prepared in DMF) in a slow-moving shaker for 30 min at 25°C.
101. Assay mixtures were mixed with 50 μM ADHP (HRP fluorogenic substrate) and transferred to a 96-well black plate.
102. Initiate DNAzyme reaction by addition of increasing the concentration of H2O2 from 30 to 800 μM.
103. Monitor the specific DNAzyme activity (oxidation reaction) fluorescently by taking readings of change in fluorescence emission spectrum at λex = 558 nm, λem = 585 nm for 3 min using the Varioskan LUX multimode microplate reader.
104. Calculate the enzyme kinetics parameters (KM and Vmax) with standard deviation from changes in the emission spectrum of triplicate values by fitting all normalised data points to the Michaelis–Menten equation using GraphPad Prism Software.
105. Calculate the DNAzyme efficiency constant using the equation lim Ef = Kcat/KM [21].
106. Further, determine the DNAzyme topologies in the presence and absence of cofactor hemin using a Jasco J-815 CD spectrometer.
107. Add 4 μM of selected DNAzyme variants (mSBDZ-X-3, PS2.M aptamer, and [B7]-3-0 aptamer) in 1× enzyme assay buffer (50 mM Tris- HCl pH 7.5, 150 mM NH4Cl, and 0.03% Triton X-100) for denaturation at 95°C for 10 min and were slowly cooled to 25°C over 1 h. **NOTE**: Always prepare negative control (buffer alone) without a template for the subtraction spectrum with test samples.
108. CD spectra were collected over a wavelength range of 340–220 nm using scanning speed at 100 nm min −1 at 25°C using a temperature-regulated cell compartment holder of 1.0 mm cell length. **NOTE**: Perform an average of at least three scanning measurements for better analysis.
109. To evaluate the DNA conformational change in the presence of a cofactor, add 12 μM of hemin.
110. Incubate samples for 30 min at 25°C.
111. Perform three scans over a 340–220 nm wavelength range using the same speed as Step 108.
112. Subtract the buffer background spectra in test samples and analyse data using GraphPad Prism Software.

## Troubleshooting

Troubleshooting advice can be found in Table 1.

**Table 1.** Troubleshooting table.

Step	Problem	Possible explanation	Solution
7, 9	Poor enzyme activity	Recycled Hemin	This is probably due to the degradation of hemin over continuous freeze and thaw if diluted in DMSO or left at room temperature for a prolonged time. Make fresh hemin in DMF before use and avoid light during preparation and use.
		Recycled ADHP	Ensure the ADHP solution does not freeze and thaw many times, and do not pre-expose it to light before/during use.
		Recycled H_2_O_2_	Make H_2_O_2_ fresh in nuclease-free water right before the enzyme assay. Don’t prepare and left for a long time at room temperature.
8	Chance of non-specific labeling	Prolonged enzyme assay	Avoid enzyme assay for a prolonged period in later selection rounds or Decrease reaction time upon increasing the selection rounds.
		Failed quenching of the enzyme reaction on time	Quench the reaction at the end time of the enzyme assay. Give at least 10–20 min or sufficient quenching time before DNA purification throughout selection.
10	Low recovery of single-stranded self-biotinylated ssDNA from enzyme assay buffer	Insufficient use of DNA binding buffer	Optimize and follow the guidelines of Zymo Research ssDNA/RNA Clean & Concentration Kit or other efficient approach for recovery of self-biotinylated ssDNA
44	Low recovery of ssDNA from PAGE	Insufficient shaking or vortexing time	Allow sufficient shaking and vortexing time for recovery of ssDNA from PAGE gel in the presence of extraction buffer.
53	Low yield PCR product later stage of selection	G-rich template	Optimize the PCR cycle number and may switch to a different buffer for G-rich amplification.

## Anticipated results

The directed evolution method outlined in this protocol can be used to evolve potential peroxidase DNAzyme/RNAzyme from a desired library. Previously, two primary strategies have been developed that focus on affinity as a selection pressure, rather than enzyme kinetics, to evolve peroxidase DNAzymes [3, 9]. This new function-based laboratory evolution workflow (Fig. 1) described here is more robust than traditional SELEX protocols. It provides enzyme kinetics as a susceptible monitoring platform for real-time detection of the DNA pool’s specific activity and captures of self-covalently modified DNA molecules across selection rounds. DNA pool catalytic activity was monitored by observing the change in the intensity of the emission spectrum (V_0_(RFU s^−1^)) of oxidized ADHP substrate through the ninth round of iterative selection. Notable, the seventh round enriched DNA pool showed the highest peroxidase activity(Fig. 2a), with *V*_0_ around six times higher than that of the G-rich initial library and approximately 1.36 times higher than the reported [B7]-3-0 aptamer [9]. Additionally, gel shift analysis confirmed that the seventh round DNA pool exhibited greater self-biotinylation activity compared to both the initial and ninth rounds (Fig. 2b) [11]. Cloning, sequence analysis, and biophysical characterization of the enriched DNA pool from the seventh round led to the discovery of a potent G-quadruplex peroxidase DNAzyme, mSBDZ-X-3 [11]. Comparative enzyme kinetic analysis revealed that, the mSBDZ-X-3 exhibits more favourable catalytic kinetics properties than all previously reported DNAzyme variations as shown by its *K*_cat_, *K*_M_, *V*_max_ and (*K*_cat_/*K*_M_) values (Fig. 3). Structural analysis revealed that the mSBDZ-X-3 DNAzyme forms parallel G-quadruplex structure. The PS2.M aptamer undergoes a structural switch from mixed type to parallel, whereas the [B7]-3-0 aptamer only partially alters its structure (Fig. 4). Additional results from dissociation constants (*K*_d_) and thermal stability analyses confirmed that the evolved mSBDZ-X-3 DNAzyme demonstrates the highest catalytic activity despite improved affinity and thermal stability compared to all previously reported peroxidase DNAzymes [11].

## Supplementary Material

bpae088_Supplementary_Data

## Data Availability

The data utilized in this study can be found in the article.

## Related link

Key reference using this protocol

*J. Am. Chem. Soc.* 2023, 145, 23, 12726–12736 https://doi.org/10.1021/jacs.3c02625
