# Supplementary material for: Directed evolution of peroxidase DNAzymes by a function-based approach
Source: Biol Methods Protoc. 2024 Dec 13;10(1):bpae088. doi: 10.1093/biomethods/bpae088 (PMC11780874; doi:10.1093/biomethods/bpae088)
Supplement: bpae088_Supplementary_Data [file bpae088_supplementary_data.pdf]

Supplementary data for

Directed evolution of peroxidase DNAzymes by a function-based approach

Soubhagya K. Bhuyan[1,2*], Weisi He[1], Jingyu Cui[1] and Julian A. Tanner[1,2,3*]

[1]School of Biomedical Sciences, LKS Faculty of Medicine, The University of Hong Kong, Hong Kong SAR, P.R. China.
[2]Advanced Biomedical Instrumentation Centre, Hong Kong Science Park, Shatin, New Territories, Hong Kong SAR, P.R. China
[3]Materials Innovation Institute for Life Sciences and Energy (MILES), HKU-SIRI, Shenzhen, P.R. China

[*] Corresponding author's email: soubhagyabhuyan@gmail.com, jatanner@hku.hk

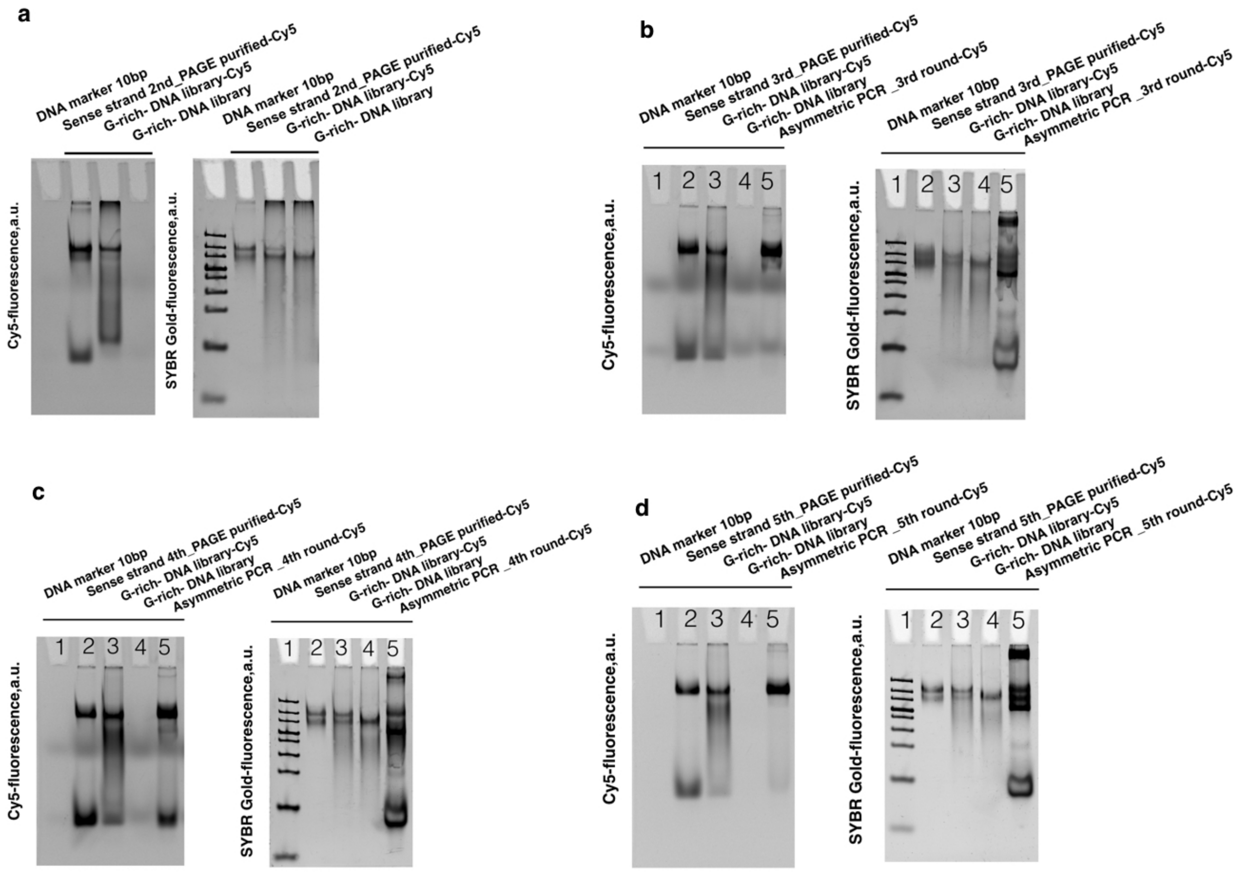

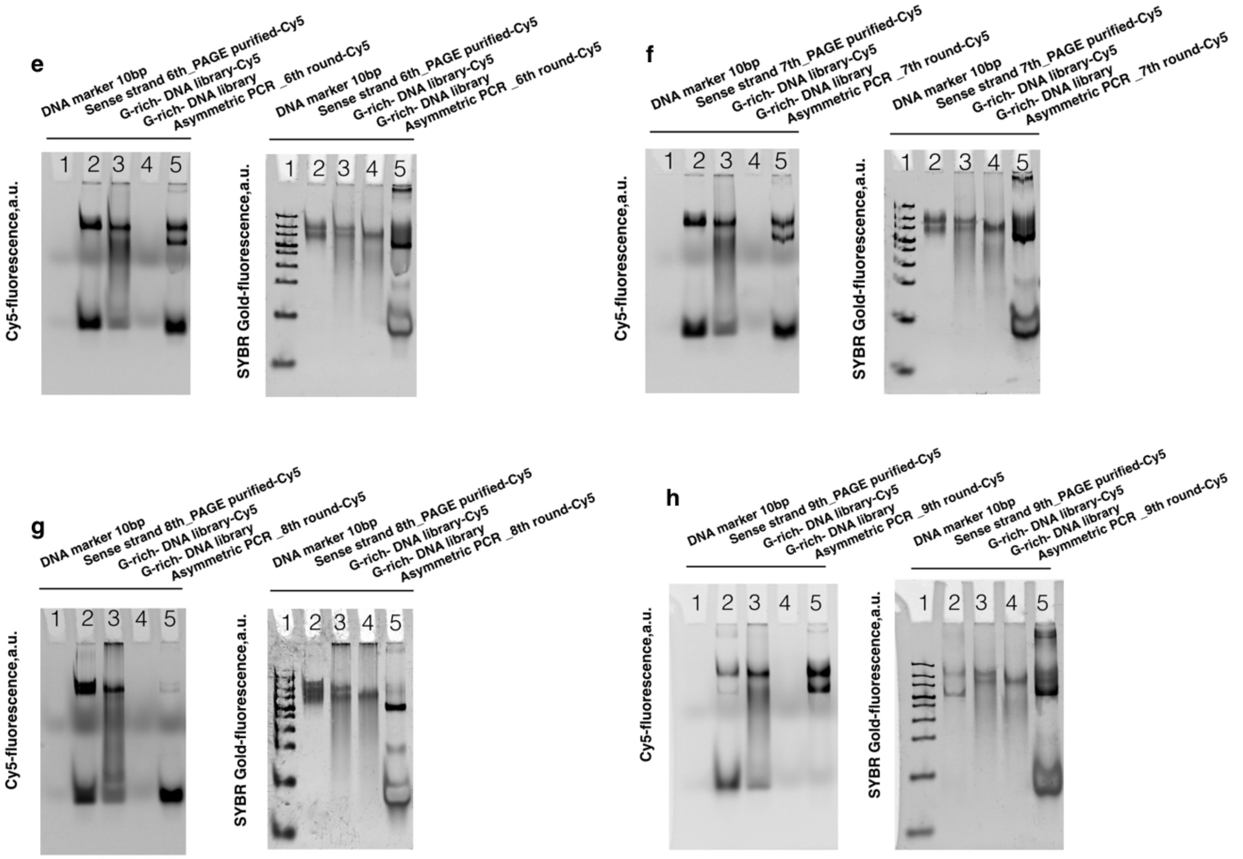

Figure S1. Qualitative assessment of PAGE-purified sense-strand DNA throughout selection rounds **(a-h)**. The Cy5-labeled reverse primer probe recognizes the sense-strand DNA in the samples based on the hybridization mechanism and ignores double-stranded DNA. The dataset was adapted with permission from Bhuyan et al(11), American Chemical Society.
